# Supplementary material for: Analysis of Cell–Cell Bridges in Haloferax volcanii Using Electron Cryo-Tomography Reveal a Continuous Cytoplasm and S-Layer
Source: Front Microbiol. 2021 Jan 13;11:612239. doi: 10.3389/fmicb.2020.612239 (PMC7838353; doi:10.3389/fmicb.2020.612239)
Supplement: Supplementary file 3 [file Data_Sheet_1.PDF]

Supplementary material to

Analysis of cell-cell bridges in *Haloferax volcanii* using Electron cryo-tomography

Shamphavi Sivabalasarma[1], Hannah Wetzel[1], Phillip Nußbaum[1], Morgan Beeby[2] and Sonja-Verena Albers[1]

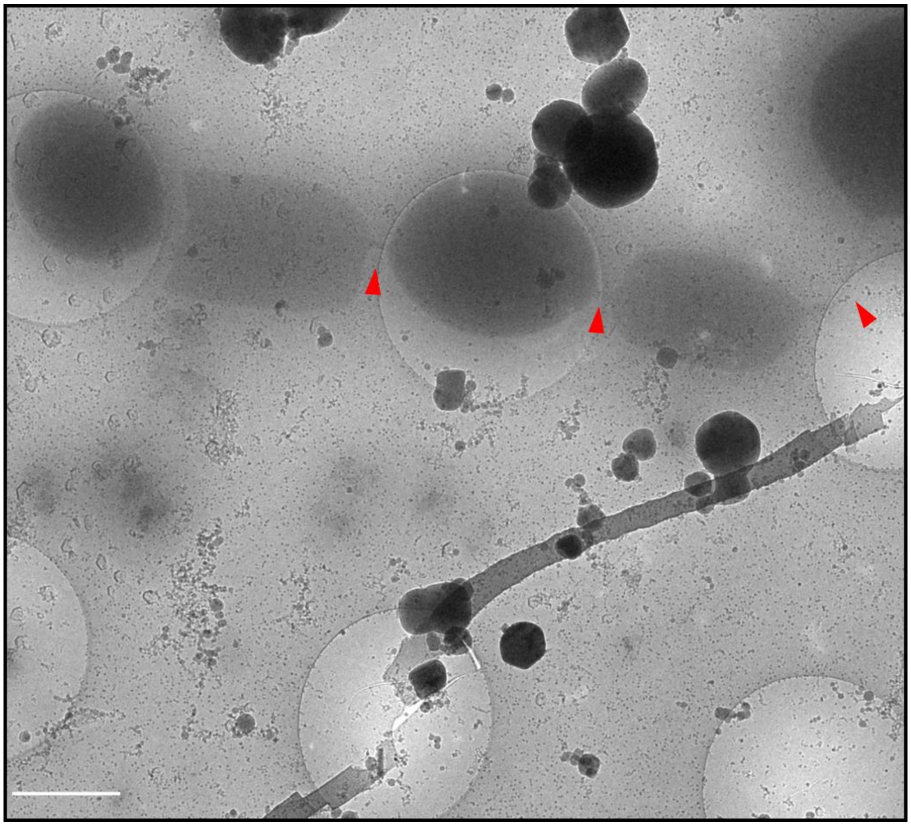

Supplementary Figure 1: Cryo-electron micrograph of a chain of cells. The cell body are connected via intercellular bridges indicated with the red arrows. Scale bar is 500 nm.

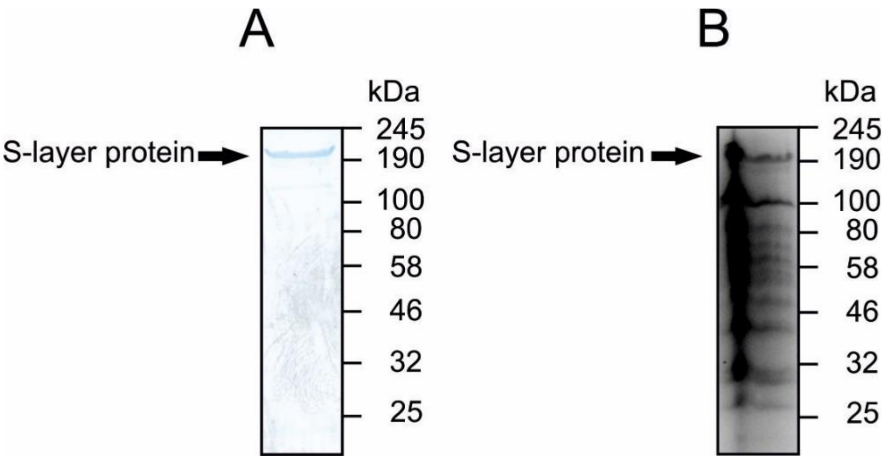

Supplementary Figure 2: SDS-PAGE and Fluorescence detection of isolated S-layer from *H. volcanii.* (A) To verify the staining of the S-layer with AlexaFluor488, S-layer was isolated. The indicated band running at 190 kDa was verified as S-layer glycoprotein via Mass spectrometry. (B) The S-layer and membrane were isolated from fluorescently stained cells and the fluorescence signal was detected. The band running at 190 kDa corresponds to (A) and can be determined as S-layer glycoprotein.

Supplementary movie 1:

Tomographic reconstruction of a cell-cell bridge. Scale bar is 100nm

Supplementary movie 2:

Fluorescence time-lapse microscopy of *H.volcanii* cells on an agar pad stained with Alexa Fluor 488 NHS Ester. Scale bar is 4 µm

Supplementary Table 1: Measured S-layer thickness and cell-cell width of selected reconstructed tomograms

Session and tomography stack number		S-Layer /nm	Average thickness S-Layer /nm	Bridge width/nm		Average bridge of width /nm	Standard error width	Length /nm
1	1	16.80	15.20	107.39	81.23	96.65	23.24	1070.96
		14.64		107.75	61.47			
		14.16		127.96	94.09			
	7	23.60	23.24	106.93	94.38	93.32	11.06	738.71
		22.90		96.14	74.63			
		23.02		99.75	88.11			
		23.42						
	20	18.73	20.46	79.63	75.56	77.10	2.96	266.94
		20.04		80.05	72.78			
		22.61		79.24	75.34			
	30	14.48	17.58	100.19	97.12	96.43	5.05	429.33
		20.70		96.93	102.73			
		17.55		88.63	92.97			
	37	22.60	19.25	82.21	85.96	86.54	7.43	1151.52
		16.30		82.10	81.49			
		18.84		101.10	86.40			
2	15	21.73	19.60	97.43	99.93	102.44	10.62	880.48

		17.83		117.01	118.44			
		19.25		92.87	108.40			
	23	17.19	19.26	90.61	65.33	78.75	17.31	2144.91
		20.69		103.77	61.26			
		19.91		86.44	65.11			
	29	24.61	22.63	79.48	76.33	71.67	8.68	265.32
		21.49		61.32	81.21			
		21.78		62.11	69.57			
	41	23.33	22.30	57.96	80.38	72.00	9.43	529.35
		21.73		72.27	72.02			
		21.85		85.77	72.43			
	56	27.12	25.71	81.70	49.05	57.63	13.33	801.29
		26.00		54.06	43.05			
		24.01		57.10	60.80			
	73	19.54	19.81	168.34	169.09	162.24	6.81	358.65
		20.24		163.55	152.62			
		19.66		154.83	161.20			
	87	18.51	18.32	98.43	98.72	96.12	4.69	955.13
		17.12		99.21	88.94			
		19.32		90.72	91.97			
3								

	67	18.86	23.18	148.05	124.85	131.46	10.54	-
		26.24		121.20	131.98			
		24.43		123.22	139.44			
	53	18.38	19.41	81.80	91.83	90.25	8.65	253.13
4		19.60		88.87	105.21			
		20.24		81.80	92.02			
	79	20.56	20.00	108.11	75.46	100.69	52.54	-
		21.85		113.27	132.93			
		17.60		163.16	11.20			
	146	19.78	20.40	71.23	67.33	71.77	3.04	570.92
		20.30		73.11	69.65			
		21.13		75.98	73.30			
5								
	6	22.80	20.66	154.64	125.70	133.71	29.82	-
		19.91		133.51	105.63			
		19.27		180.34	102.41			
	61	16.41	19.05	149.66	162.43	152.09	43.24	-
		19.53		199.80	100.85			
		21.21		196.40	103.38			
	72	19.87	20.52	69.55	62.40	69.60	1.17	463.76

		21.53		70.80	68.50			
		20.15		68.46	69.56			
	86	19.94	19.68	50.99	102.03	62.42	22.26	1059.26
		20.21		50.43	46.82			
		18.88		48.13	76.09			

Supplementary Table 2: Measures average thickness of S-layer, widths of cell-cell bridges and measured lengths of the cell-cell bridges

Average S-layer thickness/ nm	average bridge width/nm	length/nm
15.20	96.65	1070.96
23.24	93.32	738.71
20.46	77.10	266.94
17.58	96.43	429.33
19.25	86.54	1151.52
19.60	102.44	880.48
19.26	78.75	2144.91
22.63	71.67	265.32
22.30	72.00	529.35
25.71	57.63	801.29
19.81	162.24	358.65
18.32	96.12	955.13
23.18	131.46	-
19.41	90.25	253.13
20.00	100.69	-
20.40	71.77	570.92
20.66	133.71	-
19.05	152.09	-

20.52	69.60	463.76
19.68	62.42	1059.26
